# The pitfall of empathic concern with chronic fatigue after a disaster in young adults

**DOI:** 10.1186/s12888-019-2323-0

**Published:** 2019-11-04

**Authors:** Seishu Nakagawa, Motoaki Sugiura, Atsushi Sekiguchi, Yuka Kotozaki, Carlos Makoto Miyauchi, Sugiko Hanawa, Tsuyoshi Araki, Atsushi Sakuma, Ryuta Kawashima

**Affiliations:** 1 Division of Psychiatry, Tohoku Medical and Pharmaceutical University, Sendai, Japan; 2 Department of Human Brain Science, Institute of Development, Aging and Cancer (IDAC), Tohoku University, 4-1 Seiryo-machi, Aoba-ku, Sendai, 980-8575 Japan; 3 International Research Institute of Disaster Science, Tohoku University, Sendai, Japan; 4 Division of Medical Neuroimage Analysis, Department of Community Medical Support, Tohoku Medical Megabank Organization, Tohoku University, Sendai, Japan; 5 Department of Behavioral Medicine, National Institute of Mental Health, National Center of Neurology and Psychiatry, Kodaira, Tokyo, Japan; 6 Advanced Brain Science, Institute of Development, Aging and Cancer (IDAC), Tohoku University, Sendai, Japan; 7 Advantage Risk Management Co., Ltd., Tokyo, Japan; 8 Department of Psychiatry, Tohoku University Graduate School of Medicine, Sendai, Japan

**Keywords:** Chronic fatigue, Empathic concern, Disaster, Disillusionment phase, Relating to others

## Abstract

**Background:**

Empathic concern (EC) is an important interpersonal resilience factor that represents positive adaptation, such as “relating to others” (a factor of posttraumatic growth [PTG]) after disaster. However, controversy exists regarding whether the changes in EC (e.g., the intra-personal change between the acute phase and the disillusionment phase) positively or negatively affect mental health after a disaster. We hypothesized that increased EC may increase chronic fatigue due to over-adjustment (hypothesis 1). We also hypothesized that increasing the changes in “relating to others” could decrease the changes in chronic fatigue (hypothesis 2).

**Methods:**

Forty-nine young, healthy volunteers (M/F: 36/13; age at 3 months after the disaster [3 months]: mean ± SD: 21.1 ± 1.7 years) underwent assessments of EC using the Japanese version of the Interpersonal Reactivity Index, chronic fatigue using the Japanese version of the Checklist Individual Strength (CIS-J) questionnaire, and “relating to others” using the Japanese version of the PTG inventory during the acute phase (3 months) and the disillusionment phase (1 year after the disaster). Pearson product moment correlations at 3 months and 1 year were determined for all scores related to EC.

The changes (delta = degree of change from 3 months to 1 year) or scores at 1 year were entered into linear structural equation systems to test the hypotheses.

**Results:**

The delta of EC positively affected the delta of the CIS-J, and the delta of relating to others negatively affected the delta of the CIS-J. Both the EC and relating to others scores were negatively associated with the CIS-J score at 1 year. These results were in accordance with hypothesis 1 and 2.

**Conclusions:**

We demonstrated the opposite effects of 2 types of ECs, i.e., stability (inherent disposition) and flexibility (degree of change), on the degree of chronic fatigue. Increasing EC with increasing chronic fatigue, but not the change in relating to others, may be a red flag for individuals during the disillusionment phase.

## Background

Empathic concern (EC), which relates to other-oriented feelings of sympathy and concerns for unfortunate others [1], is an important emotion that enhances resilience [2]. From a longitudinal perspective, resilience is defined as the ability to recover from adversity [3] and go on with life [4]. Enhancing resilience is important for recovery from severe shocks resulting from disasters [5]. However, to our knowledge, there are two controversial issues regarding EC.

One issue is that there may be two opposing aspects of the stability of EC, i.e., that of EC as an inherent characteristic and that of its changes (flexibility). Some studies have examined inherent EC and its relation to related symptoms and statuses [6–8]. Inherent EC has been positively associated with compassion fatigue and satisfaction [6] and negatively associated with burnout among nurses in public hospitals [6] and among general practitioners [7]. To the best of our knowledge, no study has clearly described the changes (intra-personal change) of EC [7, 9], although a review demonstrated that empathic ability could be increased in nursing professionals through empathy education programmes [10].

The other issue is that EC has positive and negative effects with respect to traumatic events [11]. Regarding positive effects, EC has been related to prosocial behaviour [12], stress alleviation, and social support [13]. Furthermore, compassion, which is defined as a deep sense or awareness of the suffering of other people with the desire and empathy to relieve it [14], is a desired attitude in medical and social work practice [14, 15]. Given the health effects of disasters, positive adaptation is an inevitable aspect of preventive medicine in the face of disasters [16]. One type of positive adaptation, posttraumatic growth (PTG), is the experience of positive change that occurs as a result of the struggle with highly challenging life crises [17]. Importantly, indirect exposure to traumatic experiences (secondary post-traumatic stress) also causes symptoms that are similar to those of post-traumatic stress [18]. Accordingly, “secondary PTG” could occur through the enormous continuing distress and struggle of people who care for direct victims [18]. There are several specific terms related to secondary PTG. For example, vicarious exposure to a victim’s trauma experience leads to “vicarious PTG” [19]. Post-traumatic “relational growth” with support by mutual empathy and empowerment is frequently observed in the relatives of patients with cancer [20, 21].

Regarding the negative view, empathy confers a risk of depression [11]. In particular, compassion fatigue is a growing chronic psychological syndrome in the health care field, affecting professionals such as nurses [22], social workers [23], and family caregivers [24]. Compassion fatigue occurs when a caregiver feels overwhelmed by repeated empathic engagement with distressed clients [25] and results from knowledge of other people’s traumatic events [26]. Empathic ability is considered central to compassion fatigue [27].

After a disaster, the prevalence rates of medically unexplained symptoms seem to increase [28]. We used the degree of chronic fatigue as an index of mental health because chronic fatigue is one of the main medically unexplained symptoms after life events [28]. No study has focused on the association between the changes in EC and mental health. We focused on the disillusionment phase (1 year after a disaster), which is typified by deteriorated mental health, such as extreme fatigue, stress, and low energy [16].

We hypothesized that increasing the changes (intra-personal change) of EC may increase the changes in chronic fatigue from the acute phase to the disillusionment phase after a disaster due to over-adjustment (hypothesis 1) [11, 27]. Importantly, only when they are accompanied by reciprocal behaviours do both EC and altruism seem to become resilience factors via the enhancement of group resilience [29].

In comparison, relating to others, which is a factor of PTG that reflects human ties and mutual help [30], is an interpersonal dynamic of salutogenic change. Relating to others implies a change in subjective relationships, including a sense of EC and mutual intimacy and closeness [31]. Accordingly, we also hypothesized that increasing the changes in “relating to others” could decrease the changes in chronic fatigue (hypothesis 2).

Our purposes were to test the two hypotheses by examining the effects of the changes in EC and relating to others on chronic fatigue.

## Methods

We researched the associations among the changes (delta = degree of change between 3 months and 1 year post-disaster) in EC and relating to others as distinguished salutogenic factors, chronic fatigue as a proxy for mental health, and depression as a main confounding factor of chronic fatigue [11, 32, 33] for all hypotheses. We also researched the interpersonal aspects (via a cross-sectional analysis at 3 months and 1 year) of EC and other psychological measures.

### Subjects

We recruited Tohoku University students who had been in residence during the Great East Japan earthquake, which caused serious damage to the Tohoku area. This huge disaster had negative psychological effects on both the general population and those who were directly impacted [34].

Fifty-nine subjects (M/F: 42/17; age: mean ± SD: 21.1 ± 1.7 years) were recruited from among undergraduate and postgraduate students in the Tohoku University community 3 months after the disaster (mean days ± SD: 104 ± 9). Forty-nine (M/F: 36/13; age: mean ± SD: 21.1 ± 1.7 years) of the 59 subjects were available to participate in the research at 1 year (mean ± SD: 362 ± 18 days). The 49 subjects were in Miyagi prefecture (the disaster area) when the earthquake occurred, and they stayed there for at least 1 year. They were screened for the absence of neuropsychiatric disorders, including post-traumatic disorder (PTSD), using the Mini International Neuropsychiatric Interview (M.I.N.I.) [35, 36] at 3 months. Through the M.I.N.I., we could confirm that none of the subjects had been exposed to life-threatening trauma due to the earthquake and tsunami and that no subject had any history of psychiatric illness. However, all subjects who lived in Miyagi prefecture were strongly affected by this earthquake. For more details, see our previous study [31].

### Assessments

#### Assessment of empathy

Empathy is defined as the “reactions of one individual to the observed experiences of another” [1]. The Interpersonal Reactivity Index (IRI) [1] is the most widely used multidimensional empathy research measure [37]. We used the Japanese version of the IRI, the IRI-J [38]. The IRI-J has 4 subscales, each comprising 7 different items; the EC subscale assesses other-oriented feelings of sympathy and concern for the unfortunate, e.g., “I often have tender, concerned feelings for people less fortunate than me” [38]. The IRI-J has shown good validity in Japanese subjects [38]. The 7 items are answered on a 5-point Likert scale ranging from “Does not describe me well (4 points)” to “Describes me very well (0 points)” [1, 38].

#### Assessment of chronic fatigue

The Checklist Individual Strength (CIS) questionnaire is the most commonly used chronic fatigue questionnaire worldwide [39, 40]. Furthermore, the questionnaire has been used for both patients with chronic fatigue syndrome [41, 42] and healthy subjects [40, 43, 44]. The Japanese version of the CIS (CIS-J) comprises 20 statements and has shown good reliability and acceptable validity [44]. The total score is an index of chronic fatigue [40, 44]. A higher score indicates higher fatigue. On the CIS-J, subjects rate their perceptions of subjective symptoms over the previous 2 weeks from 1 to 7.

#### Assessment of posttraumatic growth (PTG)

The Post-traumatic Growth Inventory (PTGI) was administered by anchoring each question specifically to the earthquake [30]. The original PTGI is a 21-item scale that evaluates the subject’s success in coping with the aftermath of a trauma by measuring the degree of positive change in the individual in terms of reconstructing or strengthening perceptions of him/herself, others, and the meaning of events [30]. We used the Japanese version of the PTGI (PTGI-J) [45]. The PTGI-J has good reliability and validity [45]. In this study, a particular focus was placed on the social factor of relating to others. This factor suggests that people who are agreeable might find that others respond more supportively to them after a trauma experience than they had before [30, 45]. All items are rated on a 6-point Likert scale that ranges from 0 (*not at all*) to 5 (*to a very great degree*).

#### *Assessment of depression* (main confounding factor of chronic fatigue [46])

The Center for Epidemiologic Studies Depression Scale (CES-D) was developed to assess the epidemiology of depressive symptoms, including demonstrable sensitivity to significant life events, in the general population [47, 48]. We used the Japanese version of the CES-D, the CES-D-J [49]. The CES-D-J has shown good validity [49, 50]. It contains 20 items that are rated on a 4-point scale ranging from 0 (*rarely or never*) to 3 (*most or all of the time*).

### Analysis

We conducted two-tailed paired *t*-tests on the scores for the CIS-J, EC, relating to others as a distinct salutogenic factor, and CES-D-J scores obtained at 3 months and 1 year to show a significant distinction between the acute and disillusionment phases. Pearson product moment correlations were used to examine the relationships among the CIS-J, EC, relating to others, and CES-D-J scores to test the hypotheses. These analyses were conducted using IBM SPSS Statistics for Windows (Version 22.0).

All the change factors that made a significant independent contribution to the delta of the EC scores were entered into linear structural equation systems (AMOS 25) to explore the interrelationships among these variables (CIS-J, relating to others, and CES-D-J) and EC for the hypotheses. Because our hypotheses were based on the effect of EC on related variables, we fixed the “effects of EC” in all models. The CIS-J was affected by relating to others and the CES-D-J (model 1). The CIS-J was affected by relating to others, but it effected the CES-D-J (model 2). The CIS-J effected relating to others and the CES-D-J (model 3). Models 1 to 3 used the scores at 1 year. Models 4 to 6 used the delta.

## Results

**Fig. 1:**
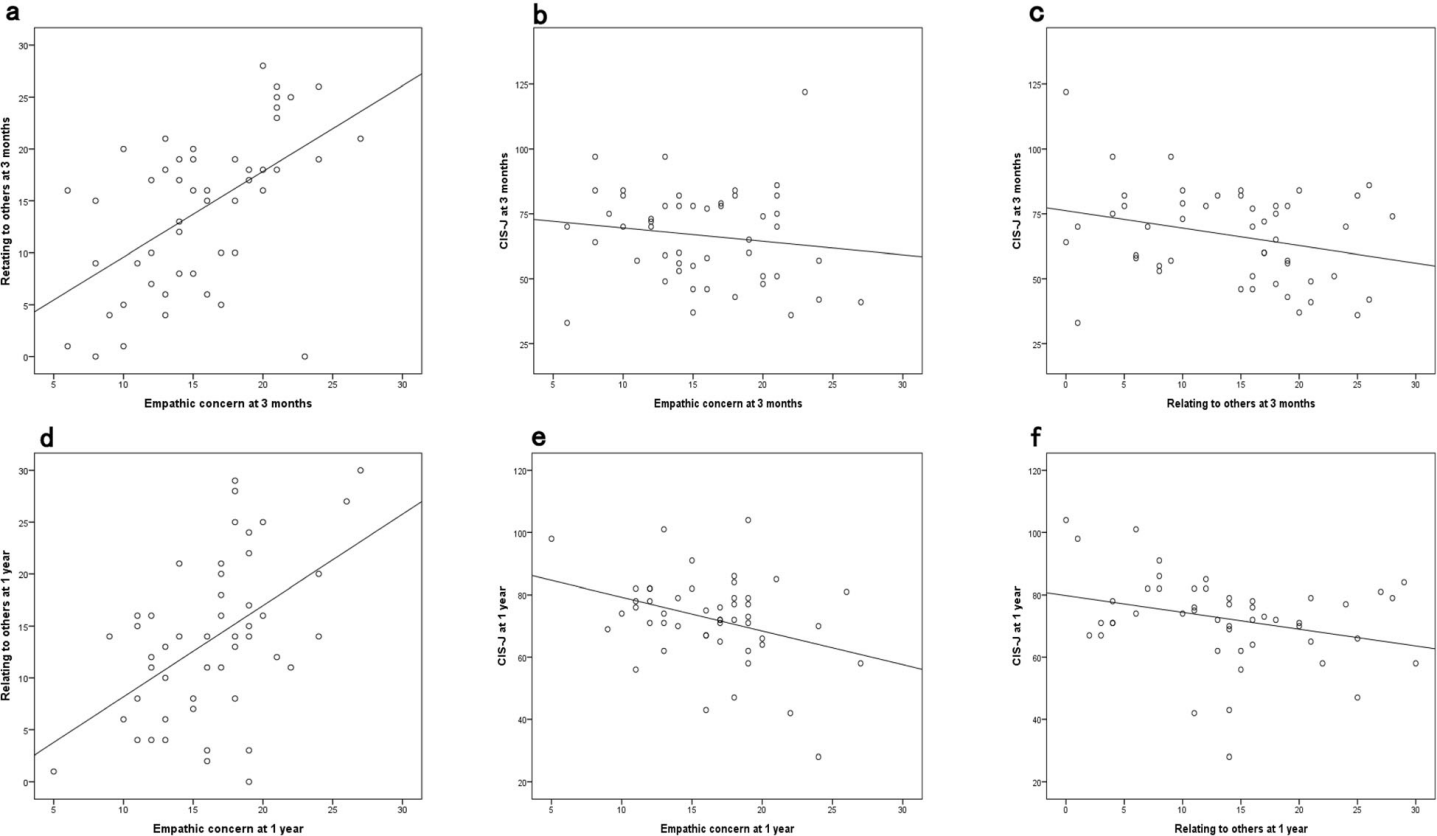
Relationships among empathy concern, relating to others, and CIS-J scores at both timepoints. Empathic concern scores were positively associated with the scores for relating to others at 3 months (**a**). Scores on the Japanese version of Checklist Individual Strength (CIS-J) were negatively related to scores for empathic concern (**b**) and relating to others (**c**) at 3 months. Empathic concern scores were positively associated with scores for relating to others at 1 year (**d**). Scores of the CIS-J scores were negatively related to scores for empathic concern (**e**) and relating to others (**f**) at 1 year

The subjects’ scores on the psychological measures at 3 months and 1 year are shown in Table 1. The two-tailed paired *t*-test (*t* [48] = − 2.30, *P* < 0.05) revealed a significant difference between the CIS-J scores at 3 months and those at 1 year (Table 1), showing the difference between the acute and disillusionment phases. Relationships among empathy concern, relating to others, and CIS-J scores illustrated by the scatter plots at 3 months and 1 year (Fig. 1). There was a significant negative association between the EC and CIS-J scores at 1 year (Table 2). A significant positive correlation was found between the scores of relating to others, as an extinguished salutogenic factor, and EC at both timepoints (Table 2).

**Table 1:** CIS-J, empathic concern, relating to others, and CES-D-J scores

	3-month (mean ± SE)	1-year (mean ± SE)	*P* value
CIS-J	66.7 ± 2.6	72.4 ± 2.0	0.026*
Empathic concern	15.6 ± 0.7	16.3 ± 0.6	0.124
Relating to others	14.2 ± 1.1	13.7 ± 1.1	0.674
CES-D-J	11.9 ± 1.3	11.7 ± 1.4	0.889

*CES-D-J* Japanese version of the Center for Epidemiologic Studies Depression Scale, *CIS-J* Japanese version of the Checklist Individual Strength questionnaire, *SE* standard error

* *P* < 0.05

**Table 2:** Correlations among the CIS-J, empathic concern, relating to others, and CES-D-J scores (3-month/1-year) (*N* = 49)

Scores	CIS-J	Empathic concern	Relating to others
Empathic concern	−0.142/− 0.340*	–	–
Relating to others	− 0.279/− 0.295*	0.550**/0.506**	–
CES-D-J	0.687**/0.208	−0.040/− 0.123	−0.045/− 0.074

*CES-D-J* Japanese version of the Center for Epidemiologic Studies Depression Scale, *CIS-J* Japanese version of the Checklist Individual Strength questionnaire

* *P* < 0.05, ** *P* < 0.01

**Fig. 2:**
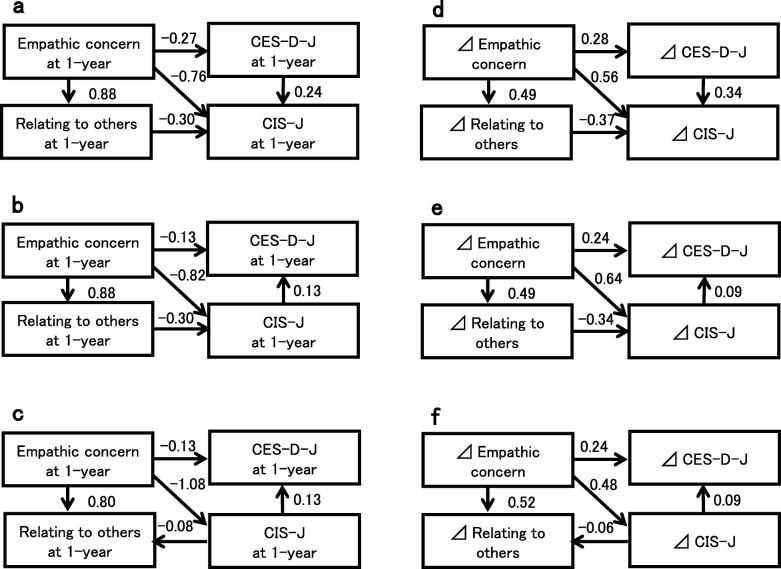
Interrelationships among scores for psychological measures at 1 year and delta. The EC scores affected all psychological measures in all models. The Japanese version of the Checklist Individual Strength (CIS-J) was affected by the scores for relating to others and the Japanese version of the Center for Epidemiologic Studies Depression Scale (CES-D-J) (model 1 (**a**)). The CIS-J was affected by relating to others but effected the CES-D-J score (model 2 (**b**)). The CIS-J affected relating to others and the CES-D-J score (model 3 (**c**)). Models 1 to 3 used the participants’ scores at 1 year. Models 4 (**d**), 5 (**e**), and 6 (**f**) used the delta. ⊿ means the delta (degree of change from 3 months to 1 year after the disaster). A one-headed arrow is used to indicate the direction of the observed regression. The numbers on the arrows represent standardized regression coefficients. Error components are omitted for simplicity

All models showed a good fit (goodness of fit [GFI] = 1, adjusted goodness of fit [AGFI] = 0.999, comparative fit index [CFI] = 1.000, and root mean square error of approximation [RMSEA] < 0.001 for Models 1 (Fig. 2a), 2 (Fig. 2b), and 3 (Fig. 2c); GFI = 0.997, AGFI = 0.973, CFI = 1.000, and RMSEA < 0.001 for Model 4 (Fig. 2d); GFI = 0.995, AGFI = 0.951, CFI = 1.000, and RMSEA < 0.001 for Models 5 (Fig. 2e) and 6 (Fig. 2f)). The delta of EC was positively related to the delta of the CIS (Fig. 2d, e, f) for Hypothesis 1, whereas the degree of EC at 1 year was negatively related to the degree of CIS-J (Fig. 2a, b, c).

## Discussion

To the best of our knowledge, this is the first study to demonstrate that the increasing changes (= delta; changing degrees from 3 months to 1 year) in EC were associated with increasing changes in chronic fatigue. These outcomes are in accordance with our hypothesis 1. The balance of EC seems to be important for stressful situations, as distress and well-being are related to empathy [33], although other-oriented empathy could enhance secondary PTG [51]. Twenty subjects in this study showed chronic fatigue based on the CIS-J total scores (chronic fatigue; more than 76) [52]. Empathy was associated with distress and anxiety in the young generation at the extreme empathy level [11, 33]. Furthermore, EC is basically a one-way emotion similar to altruism (the personality trait that leads people to care about and help others) [29]. The EC from 3 months to 1 year in this study increased in discordance with compassion fatigue because compassion fatigue is defined as the reduced capacity in being empathic [25, 26, 53]. However, we should be alert to inhabitants with increasing EC and chronic fatigue in the disillusionment phase, although they do not become compassion fatigue. An increasing number of studies have examined empathy-based stress, i.e., not only compassion fatigue but also secondary traumatic stress and vicarious traumatization [54]. In fact, after the Great East Japan earthquake, the psychological stress caused symptoms similar to those of the PTSD at subclinical and preclinical levels in inhabitants [55].

Regarding the positive effect of EC, the subjects with more EC have less chronic fatigue as resilience based on the result that there was a significant negative association between chronic fatigue and EC at 1 year. Empathic mutual relationships are basic components for promoting resilience in traumatic events [19]. EC moderated depression and general distress as resilience and has been related to well-being [32, 33]. In fact, cognitive and affective empathy may interact to protect against burnout in general practice [7], and resilience can be enhanced by increasing the empathy of medical residents [2]. Interpersonal flexibility, which has been described as an ability to adjust behaviour, seems to be central to a healthy personality for psychological adjustment and environmental pressure [56].

We should explain the results regarding hypothesis 2, i.e., increasing changes (intra-personal change) in relating to others can decrease the changes in chronic fatigue unlike EC. Relating to others was associated with adaptive and prosocial affective responses [57, 58]. PTG, e.g., relating to others, is defined as a positive psychological change, representing the result of a struggle with major life or traumatic events [30], including self-perceptions and perceptions regarding mutual relationships, to grow to a healthy level [58]. Interestingly, decreased communication was related to a greater degree of probable PTSD, depression, and distress in local workers at 20–22 months after this disaster [59]. Accordingly, it is natural that the changes in relating to others were salutogenic.

Finally, we should note a limitation of this study. Because higher intelligence may contribute to higher developmental resilience [60], these results might be limited to a well-educated young generation. The sample size (49 participants) is considered small for an epidemiological study. Further investigations using larger and more diverse samples are needed to generalize and confirm our results.

## Conclusions

We demonstrated that the effects of EC regarding the degrees at 1 year and changes (= delta; changing degrees from 3 months to 1 year) in chronic fatigue were distinct and that the decreasing changes in EC from the acute phase to the disillusionment phase may represent salutogenic active suppression to protect against increasing chronic fatigue. Relapsing/remitting, delayed dysfunction, and chronic dysfunction trajectories can be found as negative outcomes in the aftermath of a disaster [61, 62]. We should pay careful attention to the people with increasing EC and increasing chronic fatigue in the disillusionment phase after a disaster. Furthermore, a greater degree of the PTG was positively related to the degrees of confusion, anger, sadness, guilt, and anxiety in medical university student volunteers at 8 years after this earthquake [63].

## Data Availability

The datasets used and/or analysed during the current study are available from the corresponding author on reasonable request.

## Abbreviations

1 year: 1 year after the disaster
3 months: 3 months after the disaster
CES-D-J: Japanese version of the Center for Epidemiologic Studies Depression Scale
CIS-J: Japanese version of the Checklist Individual Strength
delta: degree of change from 3 months to 1 year
EC: Empathic concern
IRI-J: Japanese version of the Interpersonal Reactivity Index
M.I.N.I.: Mini International Neuropsychiatric Interview
PTG: Posttraumatic growth
PTGI-J: Japanese version of the Post-traumatic Growth Inventory
PTSD: Posttraumatic stress disorder

## References

[1] Davis MH (1983). Measuring individual differences in empathy: evidence for a multidimensional approach. J Pers Soc Psychol.

[2] Morice-Ramat A, Goronflot L, Guihard G (2018). Are alexithymia and empathy predicting factors of the resilience of medical residents in France?. Int J Med Educ.

[3] Fletcher D, Sarkar M (2013). Psychological resilience: a review and critique of definitions, concepts, and theory. Eur Psychol.

[4] Netuveli G, Wiggins RD, Hildon Z, Montgomery SM, Blane D (2006). Quality of life at older ages: evidence from the English longitudinal study of aging (wave 1). J Epidemiol Community Health.

[5] Madrigano J, Chandra A, Costigan T, Acosta JD (2017). Beyond disaster preparedness: building a resilience-oriented workforce for the future. Int J Environ Res Public Health.

[6] Duarte J, Pinto-Gouveia J, Cruz B (2016). Relationships between nurses' empathy, self-compassion and dimensions of professional quality of life: a cross-sectional study. Int J Nurs Stud.

[7] Lamothe M, Boujut E, Zenasni F, Sultan S (2014). To be or not to be empathic: the combined role of empathic concern and perspective taking in understanding burnout in general practice. BMC Fam Pract.

[8] Gross JJ, John OP (2003). Individual differences in two emotion regulation processes: implications for affect, relationships, and well-being. J Pers Soc Psychol.

[9] Everson N, Levett-Jones T, Pitt V (2018). The impact of educational interventions on the empathic concern of health professional students: a literature review. Nurse Educ Pract.

[10] Brunero S, Lamont S, Coates M (2010). A review of empathy education in nursing. Nurs Inq.

[11] Tone EB, Tully EC (2014). Empathy as a “risky strength”: a multilevel examination of empathy and risk for internalizing disorders. Dev Psychopathol.

[12] Vollhardt JR, Staub E (2011). Inclusive altruism born of suffering: the relationship between adversity and prosocial attitudes and behavior toward disadvantaged outgroups. Am J Orthop.

[13] Park KH, Kim DH, Kim SK, Yi YH, Jeong JH, Chae J, Hwang J, Roh H (2015). The relationships between empathy, stress and social support among medical students. Int J Med Educ.

[14] Perez-Bret E, Altisent R, Rocafort J (2016). Definition of compassion in healthcare: a systematic literature review. Int J Palliat Nurs.

[15] Gelhaus P (2012). The desired moral attitude of the physician: (II) compassion. Med Health Care Philos.

[16] Math SB, Nirmala MC, Moirangthem S, Kumar NC (2015). Disaster management: mental health perspective. Indian J Psychol Med.

[17] Tedeschi RG, Calhoun LG (2004). Posttraumatic growth: conceptual foundations and empirical evidence. Psychol Inq.

[18] Cieslak R, Benight CC, Rogala A, Smoktunowicz E, Kowalska M, Zukowska K, Yeager C, Luszczynska A (2016). Effects of internet-based self-efficacy intervention on secondary traumatic stress and secondary posttraumatic growth among health and human services professionals exposed to indirect trauma. Front Psychol.

[19] Nuttman-Shwartz O (2015). Shared resilience in a traumatic reality: a new concept for trauma workers exposed personally and professionally to collective disaster. Trauma Violence Abuse.

[20] Bekteshi V, Kayser K (2013). When a mother has cancer: pathways to relational growth for mothers and daughters coping with cancer. Psycho Oncol.

[21] Threader J, McCormack L (2016). Cancer-related trauma, stigma and growth: the ‘lived’ experience of head and neck cancer. Eur J Cancer Care.

[22] Jenkins B, Warren NA (2012). Concept analysis: compassion fatigue and effects upon critical care nurses. Crit Care Nurs Q.

[23] Bourassa DB (2009). Compassion fatigue and the adult protective services social worker. J Gerontol Soc Work.

[24] Lynch SH, Lobo ML (2012). Compassion fatigue in family caregivers: a Wilsonian concept analysis. J Adv Nurs.

[25] Figley CR (2002). Compassion fatigue: psychotherapists' chronic lack of self care. J Clin Psychol.

[26] Figley CR (1995). Compassion fatigue as secondary traumatic stress disorder: An overview. Compassion fatigue: Coping with secondary traumatic stress disorder in those who treat the traumatized.

[27] Sabo B (2011). Reflecting on the concept of compassion fatigue. Online J Issues Nurs.

[28] van den Berg B, Grievink L, Yzermans J, Lebret E (2005). Medically unexplained physical symptoms in the aftermath of disasters. Epidemiol Rev.

[29] Sugiura M, Sato S, Nouchi R, Honda A, Abe T, Muramoto T, Imamura F (2015). Eight personal characteristics associated with the power to live with disasters as indicated by survivors of the 2011 great East Japan earthquake disaster. PLoS One.

[30] Tedeschi RG, Calhoun LG (1996). The posttraumatic growth inventory: measuring the positive legacy of trauma. J Trauma Stress.

[31] Nakagawa S, Sugiura M, Sekiguchi A, Kotozaki Y, Araki T, Hanawa S, Makoto Miyauchi C, Sakuma A, Kawashima R (2014). Fatigue and relating to others 3 months after the 2011 great East Japan earthquake. Psychiatry Res.

[32] Cristea IA, Legge E, Prosperi M, Guazzelli M, David D, Gentili C (2014). Moderating effects of empathic concern and personal distress on the emotional reactions of disaster volunteers. Disasters.

[33] Thomas MR, Dyrbye LN, Huntington JL, Lawson KL, Novotny PJ, Sloan JA, Shanafelt TD (2007). How do distress and well-being relate to medical student empathy? A multicenter study. J Gen Intern Med.

[34] Kyutoku Y, Tada R, Umeyama T, Harada K, Kikuchi S, Watanabe E, Liegey-Dougall A, Dan I (2012). Cognitive and psychological reactions of the general population three months after the 2011 Tohoku earthquake and tsunami. PLoS One.

[35] Sheehan DV, Lecrubier Y, Sheehan KH, Amorim P, Janavs J, Weiller E, Hergueta T, Baker R, Dunbar GC (1998). The Mini-International Neuropsychiatric Interview (M.I.N.I.): the development and validation of a structured diagnostic psychiatric interview for DSM-IV and ICD-10. J Clin Psychiatry.

[36] Otsubo T, Tanaka K, Koda R, Shinoda J, Sano N, Tanaka S, Aoyama H, Mimura M, Kamijima K (2005). Reliability and validity of Japanese version of the mini-international neuropsychiatric interview. Psychiatry Clin Neurosci.

[37] Murphy BA, Costello TH, Watts AL, Cheong YF, Berg JM, Lilienfeld SO. Strengths and weaknesses of two empathy measures: a comparison of the measurement precision, construct validity, and incremental validity of two multidimensional indices. Assessment. 2018:1073191118777636. 10.1177/1073191118777636 [Epub ahead of print]. 10.1177/1073191118777636 29847994

[38] Sakurai S (1988). The Relationship between Empathy and Helping Behavior in College Students. Bull Nara Univ Educ.

[39] Vercoulen JH, Swanink CM, Fennis JF, Galama JM, van der Meer JW, Bleijenberg G (1994). Dimensional assessment of chronic fatigue syndrome. J Psychosom Res.

[40] Beurskens AJ, Bultmann U, Kant I, Vercoulen JH, Bleijenberg G, Swaen GM (2000). Fatigue among working people: validity of a questionnaire measure. Occup Environ Med.

[41] Prins JB, Bleijenberg G, Bazelmans E, Elving LD, de Boo TM, Severens JL, van der Wilt GJ, Spinhoven P, van der Meer JW (2001). Cognitive behaviour therapy for chronic fatigue syndrome: a multicentre randomised controlled trial. Lancet.

[42] Bleijenberg G, van der Meer JW, The GK (2007). The effect of acclydine in chronic fatigue syndrome: a randomized controlled trial. PLoS Clin Trials.

[43] Lee YC, Chien KL, Chen HH (2007). Lifestyle risk factors associated with fatigue in graduate students. J Formos Med Assoc.

[44] Aratake Y, Tanaka K, Wada K, Watanabe M, Katoh N, Sakata Y, Aizawa Y (2007). Development of Japanese version of the checklist individual strength questionnaire in a working population. J Occup Health.

[45] Taku K, Calhoun LG, Tedeschi RG, Gil-Rivas V, Kilmer RP, Cann A (2007). Examining posttraumatic growth among Japanese university students. Anxiety Stress Coping.

[46] Norheim KB, Jonsson G, Omdal R (2011). Biological mechanisms of chronic fatigue. Rheumatology (Oxford).

[47] Radloff L (1977). The CES-D scale: a self-report depression scale for research in the general population. Appl Psychol Meas.

[48] Shima S, Shikano T, Kitamura T, Asai M (1985). New self-rated scale for depression. Japanese J Clin Psychiatry.

[49] Shima S (1985). A new self-rating scale for depression. Seishin Igaku.

[50] Wada K, Tanaka K, Theriault G, Satoh T, Mimura M, Miyaoka H, Aizawa Y (2007). Validity of the Center for Epidemiologic Studies Depression Scale as a screening instrument of major depressive disorder among Japanese workers. Am J Ind Med.

[51] Nagamine M, Shigemura J, Fujiwara T, Waki F, Tanichi M, Saito T, Toda H, Yoshino A, Shimizu K (2018). The relationship between dispositional empathy, psychological distress, and posttraumatic stress responses among Japanese uniformed disaster workers: a cross-sectional study. BMC Psychiatry.

[52] Bültmann U, de Vries M, Beurskens AJ, Bleijenberg G, Vercoulen JH, Kant I (2000). Measurement of prolonged fatigue in the working population: determination of a cutoff point for the checklist individual strength. J Occup Health Psychol.

[53] Boscarino JA, Figley CR, Adams RE (2004). Compassion fatigue following the September 11 terrorist attacks: a study of secondary trauma among New York City social workers. Int J Emerg Ment Health.

[54] Rachel S, Rauvola D, VegaKristi N. Compassion fatigue, secondary traumatic stress, and vicarious traumatization: a qualitative review and research agenda. Occup Health Sci. 2019;3(3):297–336.

[55] Matsumoto K, Sakuma A, Ueda I, Nagao A, Takahashi Y (2016). Psychological trauma after the great East Japan earthquake. Psychiatry Clin Neurosci.

[56] Paulhus DL, Martin CL (1988). Functional flexibility: a new conception of interpersonal flexibility. J Pers Soc Psychol.

[57] Eisenberg N, Miller PA (1987). The relation of empathy to prosocial and related behaviors. Psychol Bull.

[58] Meyerson DA, Grant KE, Carter JS, Kilmer RP (2011). Posttraumatic growth among children and adolescents: a systematic review. Clin Psychol Rev.

[59] Ueda I, Sakuma A, Takahashi Y, Shoji W, Nagao A, Abe M, Suzuki Y, Matsuoka H, Matsumoto K (2017). Criticism by community people and poor workplace communication as risk factors for the mental health of local welfare workers after the great East Japan earthquake: a cross-sectional study. PLoS One.

[60] Sameroff AJ, Rosenblum KL (2006). Psychosocial constraints on the development of resilience. Ann N Y Acad Sci.

[61] Norris FH, Tracy M, Galea S (2009). Looking for resilience: understanding the longitudinal trajectories of responses to stress. Soc Sci Med.

[62] Galatzer-Levy IR, Huang SH, Bonanno GA (2018). Trajectories of resilience and dysfunction following potential trauma: a review and statistical evaluation. Clin Psychol Rev.

[63] Kaye-Kauderer HP, Levine J, Takeguchi Y, Machida M, Sekine H, Taku K, Yanagisawa R, Katz C (2019). Post-traumatic growth and resilience among medical students after the march 2011 disaster in Fukushima, Japan. Psychiatr Q.

